# Challenges in clinical dental education during COVID-19 crisis

**DOI:** 10.1186/s42506-021-00072-3

**Published:** 2021-05-24

**Authors:** Mohamed G. Hassan, Reham Hassan

**Affiliations:** 1 Department of Orthodontics, Faculty of Oral and Dental Medicine, South Valley University, Qena, Egypt; 2 Department of Orthodontics, Faculty of Dentistry, October 6 University, Giza, Egypt; 3 Department of Endodontics, Faculty of Dentistry, Minia University, Minia, Egypt; 4 Department of Endodontics, Faculty of Dentistry, Egyptian Russian University, Cairo, Egypt

## Main text

Coronavirus pandemic has affected all medical institutions, and dental schools are no exception [1]. Dentists including dental students have been considered as a very high-risk group by the Occupational Safety and Health Administration (OSHA) due to the potential for exposure to SARS-CoV-2 through aerosol-generating clinical procedures [2]. The transmission mode of SARS-CoV-2 (the causative agent of COVID-19) via respiratory droplets exposes dental healthcare providers to a high risk of infection. For this reason, many of the dental academic institutions have either closed temporarily or applied localized closures during the early wave of the pandemic. The reaction toward the COVID-19 crisis varied observing the available resources, institutional settings, and national (local) safety guidelines. Dental education has been affected given the application of various response strategies, including but not limited to transitioning from in-person classes into distant learning, changing the policies toward the in-patient care, canceling and modifying clinical rotations, professional meetings, and finally rescheduling of licensure examinations [3].

Academic dental schools are known to be demanding and highly competitive learning environments. COVID-19 pandemic highlighted the differences between medical and dental institutions in the practical and clinical educational activities. Academic dental institutions are known as competitive and demanding learning communities, where students study preclinical and clinical dental courses designed to equip them with the essential knowledge and skills for licensure and clinical practice [4]. Clinical dental interventions require the integration of students’ technical and intellectual skills which require clinically oriented education in both basic medical and dental sciences, in addition to refined practice-based education in clinical dental care.

As we are starting the third wave of COVID-19 pandemic, dental academic institutions in Egypt must learn from previous experiences and take the professional responsibility to share information. We hope that dental schools will be able to maintain adequate training in a safe clinical setting. Regarding clinical training, it is important to answer many questions that may help in understanding our future actions like are dental schools considered among high-risk workplaces or they should be treated like other educational in-campus settings? What will be the basic infection control standards? Are there any plans regarding the COVID-19 vaccination? Are there expected modifications on the future designs and infrastructure of educational dental settings including waiting areas, and spacing of dental units, and the central air ventilation? Although hybrid learning will probably be a keystone of future dental education, it is crucial to study extensively the possible pedagogical effects of the sudden change in dental educational strategies caused by the COVID-19 crisis. Finally, as an educator, I wish to go back soon to normal interactive dental education, but I believe that dental education might change significantly in the near future.

## Data Availability

The materials are available upon request.

## Abbreviations

OSHA: Occupational Safety and Health Administration
SARS-CoV-2: Coronavirus

## References

[1] Quinn B, Field J, Gorter R, Akota I, Manzanares M-C, Paganelli C (2020). COVID-19: the immediate response of european academic dental institutions and future implications for dental education. Eur J Dent Educ..

[2] U.S. Department of LaborOccupational Safety and Health Administration. Guidance on Preparing Workplaces for COVID-19. OSHA 3990-03 2020. [Internet]. [cited 2020 Dec 30]. Available from: https://www.osha.gov/Publications/OSHA3990.pdf

[3] Bennardo F, Buffone C, Fortunato L, Giudice A (2020). COVID-19 is a challenge for dental education-a commentary. Eur J Dent Educ..

[4] Akinkugbe AA, Garcia DT, Smith CS, Brickhouse TH, Mosavel M. A descriptive pilot study of the immediate impacts of COVID-19 on dental and dental hygiene students’ readiness and wellness. J Dent Educ. 2020;85(3):401–10. 10.1002/jdd.12456 PMC8043566 33084054

